# Preventing Sleep Disruption With Bright Light Therapy During Chemotherapy for Breast Cancer: A Phase II Randomized Controlled Trial

**DOI:** 10.3389/fnins.2022.815872

**Published:** 2022-03-09

**Authors:** Michelle Rissling, Lianqi Liu, Shawn D. Youngstedt, Vera Trofimenko, Loki Natarajan, Ariel B. Neikrug, Neelum Jeste, Barbara A. Parker, Sonia Ancoli-Israel

**Affiliations:** ^1^Department of Psychiatry, University of California, San Diego, San Diego, CA, United States; ^2^Department of Medicine, University of California, San Diego, San Diego, CA, United States; ^3^Edson College of Nursing and Health Innovation, Arizona State University, Phoenix, AZ, United States; ^4^St. Joseph Hospital, Orange, CA, United States; ^5^Family Medicine and Public Health, University of California, San Diego, San Diego, CA, United States; ^6^Moores Cancer Center, University of California, San Diego, San Diego, CA, United States; ^7^Department of Psychiatry and Human Behavior, University of California, Irvine, Irvine, CA, United States; ^8^Johnson & Johnson, San Diego, CA, United States

**Keywords:** breast cancer, light therapy, sleep, actigraphy, PSQI, activity

## Abstract

**Purpose:**

The goal of this study was to examine whether daily increased morning light exposure would maintain or improve sleep and the circadian pattern of relatively more activity in the day and less during the night in women undergoing chemotherapy for breast cancer.

**Patients and Methods:**

Participants were 39 women with newly diagnosed breast cancer, randomized to either 30-mins of daily morning bright white light (BWL) or dim red light (DRL). Sleep/wake was measured objectively for 72-h with wrist actigraphy and subjectively with the Pittsburgh Sleep Quality Index (PSQI) prior to and during chemotherapy cycles 1 and 4. The study was registered with the National Institutes of Health ClinicalTrials.gov (Clinical Trials number: NCT00478257).

**Results:**

Results from actigraphy suggested that compared to the DRL group, women in the BWL group had longer night-time sleep, fewer sleep disturbances during the night, and had fewer and shorter daytime naps at the end of cycle 4 of chemotherapy as well as exhibiting less activity at night and more activity during the day by the end of cycle 4. Results from PSQI indicated that components of sleep quality improved but daytime dysfunction deteriorated during cycle 4 treatment in the BWL group; meanwhile the DRL group used more sleep medications in the treatment weeks which might have led to the improved sleep quality during the recovery weeks of both cycles.

**Conclusion:**

These results suggest that bright white light therapy administered every morning on awakening may protect women undergoing chemotherapy for breast cancer from nighttime sleep and daytime wake disruption. Randomized clinical trials in larger samples are needed to confirm these findings.

## Introduction

Disturbed sleep is one of the most common and distressing complaints among patients with breast cancer, occurring in 30–50% of patients undergoing chemotherapy (Savard and Morin, 2001). Nighttime sleep disruptions, such as difficulty falling asleep, staying asleep, and frequent awakenings, are aggravated in women with breast cancer undergoing chemotherapy (Ancoli-Israel et al., 2006; Berger et al., 2007; Palesh et al., 2010). Patients with cancer also complain of increased daytime napping (Engstrom et al., 1999) described as longer and more frequent daytime naps as treatment progresses (Berger and Farr, 1999; Young-McCaughan et al., 2003; Levin et al., 2005; Wielgus et al., 2009), which has been associated with decreased daytime activity that, in turn, has been found to predict higher cancer-related fatigue (CRF) (Berger and Farr, 1999; Wielgus et al., 2009).

Many studies measuring sleep in cancer have used actigraphs, a wrist worn device which measures activity which can be used to estimate sleep and wake. Despite the ability of actigraphy to simultaneously measure both sleep and activity (Berger et al., 2008), relatively few studies have evaluated both outcomes in patients with breast cancer undergoing chemotherapy (Young-McCaughan et al., 2003; Berger et al., 2007).

Previous research in our laboratory found that women with breast cancer have decreased daytime light exposure both before and during chemotherapy, with the most pronounced decrease in light exposure during the treatment infusion weeks of chemotherapy (Liu et al., 2005). Synchronized endogenous circadian activity rhythms are related to exposure to diurnal bright light (Kripke et al., 2007); low diurnal illumination levels have been associated with nocturnal sleep dysfunction (Terman et al., 1995; Ancoli-Israel et al., 2002). Sleep and mood disruptions have been successfully treated with morning exposure to increased artificial bright light in other populations, including individuals with winter depression (Rosenthal et al., 1985; Terman and Terman, 2005), non-seasonal depression (Al-Karawi and Jubair, 2016), anxiety (Youngstedt and Kripke, 2007), and PTSD (Youngstedt et al., 2021). Our laboratory has shown that morning bright light therapy prevents cancer related fatigue from getting worse, prevents circadian activity rhythms from deteriorating and improves quality of life in women undergoing chemotherapy for breast cancer (Ancoli-Israel et al., 2011; Neikrug et al., 2012; Jeste et al., 2013). Morning light therapy has been combined with cognitive behavioral therapy to improve sleep in women undergoing chemotherapy (Bean et al., 2020); however, there are no studies evaluating just bright light therapy on sleep or activity in this group. Thus, we evaluated whether administration of bright light upon awakening in the morning would alleviate the poor nighttime sleep and lower daytime alertness experienced during chemotherapy in women with breast cancer.

## Materials and Methods

We conducted a small phase II randomized clinical pilot study comparing bright white light (BWL) therapy to dim red light (DRL) therapy in women diagnosed with breast cancer undergoing four cycles of adjuvant or neo-adjuvant chemotherapy. The study was conducted between July 2005 and June 2007

### Patients

**FIGURE 1:**
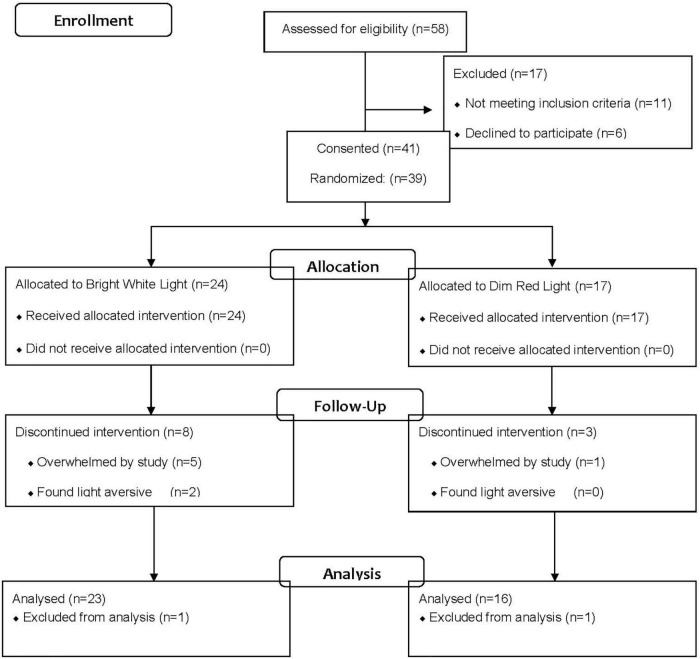
Consort table depicting sample sizes at each point of the study.

Data were collected from the same women reported in previous publications on the effect of light on fatigue, circadian activity rhythms and quality of life (Ancoli-Israel et al., 2011; Neikrug et al., 2012; Jeste et al., 2013). As reported in those studies, 58 women were referred by physicians for the study (see Figure 1). Of those referred, 17 were ineligible after screening and 41 were consented and randomized. Of the 41 randomized, two participants (one from each group) dropped out immediately and were not included in the analysis; eight women from the BWL and three women from the DRL dropped during the treatment phase and were included in the analysis. Therefore, data are presented from 39 women (mean age = 53.95 years, *SD* = 9.06, range = 32–70 years).

### Inclusion and Exclusion Criteria

Participants were referred by medical oncologists in the San Diego community or from the UCSD Moores Cancer Center. Inclusion criteria were having a new diagnosis of stage I–III breast cancer and scheduled to receive at least four cycles of adjuvant or neoadjuvant chemotherapy. Exclusion criteria were being pregnant, having metastatic or IIIB (including inflammatory) breast cancer, significant pre-existing anemia, or confounding underlying medical illnesses or any other physiological or psychological impairments that would have limited participation. Breast cancer disease staging was based on the American Joint Committee on Cancer Staging Manual 5th Edition (Greene, 2002). Menopausal status was determined using self-report of the occurrence of menses (Rissling et al., 2011).

After referral from the oncologist, informed consent, HIPAA, and release of information were obtained by the study coordinator. Pertinent medical information [e.g., stage of disease and estrogen/progesterone receptor status (ER/PR)] was abstracted from each participant’s medical record prior to participation in the study.

Approval for this study was received from the University of California San Diego Office of IRB Administration and by the UC San Diego Moores Cancer Center’s Protocol Review and Monitoring Committee. All women provided written informed consent before participation. The study was registered with the National Institutes of Health ClinicalTrials.gov (Clinical Trials number: NCT00478257).

### Study Design

**FIGURE 2:**
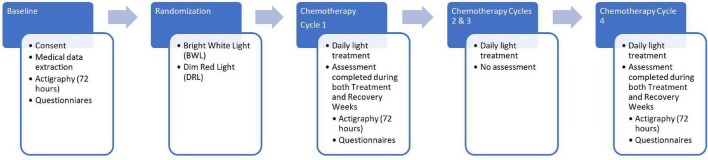
Flow diagram depicting study procedures including timing of actigraphy and questionnaire assessments.

Figure 2 shows the study design which included a baseline assessment, treatment randomization prior to the start of chemotherapy followed by daily morning light treatment for four cycles of chemotherapy. After baseline, actigraphy and questionnaires were repeated only during the treatment and recovery weeks of cycles 1 and 4 of chemotherapy. Each chemotherapy cycle was either 2 or 3 weeks as the recommended chemotherapy regimen changed in the middle of our study. Wrist actigraphs were worn for three consecutive 24-h periods (72-h) at each of the five time-points: prior to the start of chemotherapy (baseline), chemotherapy treatment week of cycle 1 (C1TW), recovery week of cycle 1 (C1RW), chemotherapy treatment week of cycle 4 (C4TW), and recovery week of cycle 4 (C4RW). Actigraphy periods coincided with each participant’s scheduled weekday chemotherapy infusions. All questionnaires could be filled out any time during the 3 days that actigraphy data were collected. The actigraph and the questionnaires were all picked up together.

Results of questionnaire data assessing fatigue, mood, quality of life, functional outcome, menopausal status and climacteric symptoms have been previously published (Liu et al., 2009, 2012; Ancoli-Israel et al., 2011; Rissling et al., 2011; Neikrug et al., 2012; Jeste et al., 2013).

#### Randomization

The randomization sequence was generated by the study statistician using the R statistical software package.^1^ A blocked design with a 2:3 allocation to dim red light (DEL; *n* = 16) versus bright white treatment (BWL; *n* = 23) using a block size of 4. Our hypothesis was that BWL would be more beneficial; and therefore, more participants were randomized to BWL to provide a larger sample with this treatment. Both treatments were non-invasive. The study coordinators were blinded to the randomization allocation of participants.

#### Instructions to Participants

Each participant was provided with a Litebook^®^, a demonstration of proper operation, a paper tape measure and digital timer. Participants were instructed how to position the Litebook^®^ and to operate it for 30 continuous minutes immediately upon awakening every day throughout their four cycles of chemotherapy. The goal of the study was described to participants by the study coordinators as an evaluation of two frequencies of light therapy (red or white) for improving sleep and fatigue during chemotherapy.

#### Light

Light was administered *via* a Litebook^®^ 1.2 (Litebook Ltd., Medicine Hat, AB, Canada). The Litebook^®^ is a small (6′′ × 5′′ × 1′′) and lightweight (8 oz.) light box designed to be placed on a table about 18′′ from the patient’s head and within a 45° visual field. As previously published (Ancoli-Israel et al., 2011; Neikrug et al., 2012), light was administered with the Litebook^®^ 1.2 (Litebook Ltd., Medicine Hat, AB, Canada). The Litebook^®^ utilizes 60 white light-emitting diode (LED) lights with a distribution of energy particularly concentrated in the middle and long wavelengths (Desan et al., 2007) and which mimic the visible spectrum of sunlight (about 1,500 lux) for minimum glare and maximum eye comfort, without emitting ultraviolet (UV) light. Two women randomized into the BWL group reported the light aversive and dropped out during treatment; however, these data are included in the analysis. An identical-appearing device utilizing red LEDs emitting dim red light at <50 lux was used for the comparison DRL group. No participants reported the dim red light aversive.

The Litebooks^®^ were modified to include an integrated meter which allowed for monitoring treatment adherence by recording operation time and duration. Partial adherence data were available for 30 participants (BWL *n* = 17; DRL *n* = 13); analysis indicated similar frequency of use (BWL = 55.0%; DRL = 70.2% of days assigned) and duration of use [BWL = 31.5 min (SD = 9.89); DRL = 33.9 min (SD = 10.93) per day used] with no significant difference between the groups.

### Measures

#### Pittsburgh Sleep Quality Index

Sleep quality was measured with the Pittsburgh Sleep Quality Index (PSQI), a 19-item questionnaire which rates patients’ reports of sleep quality, sleep latency, sleep duration, habitual sleep efficiency, sleep disturbances, use of sleep medication and daytime dysfunction (Buysse et al., 1989). The total PSQI scores range from 0 to 21 with high scores reflecting poor sleep quality. A total score above 5 is generally considered poor sleep. Due to the timeline of the data collection, in consultation with Dan Buysse, the developer of the PSQI (personal communication), the PSQI was modified to assess sleep over the past week.

#### Actigraphy

Wrist actigraphy devices were used for obtaining objective measures of nighttime and daytime sleep as well as for activity levels. Wrist actigraphy measures motion over time by recording the amount of electrical deflection during a fixed interval (e.g., minute by minute) (Ancoli-Israel et al., 2003; Ancoli-Israel et al., 2015). In the current study, two similar actigraphy devices were used. The Actillume^®^ was used with the first 11 participants (Ambulatory Monitoring, Inc., Ardsley, NY, United States). Actillume^®^ data were analyzed using the Action-3 software program (Ambulatory Monitoring Inc., Ardsley, NY, United States). Actillume^®^ data for nine participants (BWL = 5; DRL = 4) are included in these analyses. The Actiwatch-Light^®^ (Mini-Mitter| Respironics/Philips, Eindhoven, Netherlands) was used in the remainder of the participants (*n* = 28). Actiwatch-Light^®^ data were analyzed using the Actiware^®^ 5 sleep and activity monitoring software program (Mini-Mitter| Respironics). Activity sensitivity threshold was set to medium. Both devices record continuous acceleration data on the non-dominant wrist using a battery-operated microprocessor that senses motion with a piezoelectric beam and detects movement in all three axes. As previously published, device equivalency was evaluated by comparing data collected by paired devices worn simultaneously for 72-h by eight healthy adult volunteers (Liu et al., 2005, 2013a,b; Ancoli-Israel et al., 2014). The software-scored sleep/wake data based on the two types of activity count were highly correlated (both *r’s* > 0.85, both *p*’s < 0.0001), therefore, these variables were deemed equivalent for the purpose of this study. Data from 39 participants (BWL = 23, DRL = 16) are included in analyses. Actigraphic sleep variables were derived from a mean of three continuous sleep and wake (night/day) periods using 1-min epochs. Self-report *via* sleep log was used to edit actigraphy data and determine daytime and nighttime sleep and wake periods. Nighttime variables included: nighttime average activity counts per minute, sleep percentage (%sleep), nighttime total sleep time (TST) and nighttime total waketime (TWT). Daytime variables included: daytime average activity counts per minute, mean nap duration (mNAP), number of daytime naps (nNAP), and total nap time (TNT). A daytime sleep episode, or nap, was defined as any period of 10 or more minutes of consecutive actigraphic inactivity (i.e., sleep) during the period between final out of bedtime in the morning and into bedtime the following night.

### Statistical Analyses

Descriptive statistics were calculated for the entire sample as well as separately for the two treatment groups. Group differences were assessed with *t*-tests at baseline for possible confounders (i.e., demographic variables, clinical characteristics, and chemotherapy regimen). Variables that significantly differed between the treatment groups at a 0.05 significance level were controlled for in the inferential analysis.

Linear mixed-effects models were used for analyzing changes of subjective sleep quality, objective activity count and sleep/wake variables before and during chemotherapy, with group, time and group-by-time interaction included as fixed covariate effects. Baseline was the reference time point and the DRL group was the reference group. Each of the outcome variables were modeled separately. If a significant group, time or group-x-time interaction was found, further *post hoc* tests were conducted using appropriate contrasts: between group differences at each time point, and/or within group changes from Baseline to the other time points. Linear mixed-effects models and restricted maximum likelihood methods (Diggle et al., 1996) were employed for analyzing and comparing sleep and activity variables for each treatment group. This paradigm relies on the “missing at random” assumption (Diggle et al., 1996) and allows for modeling partial data where the number of measures per person could vary and participants with missing time points could still be included in the analysis. Thus, mixed model protects from a “completers only” bias.

## Results

### Demographics

Table 1 shows the sociodemographic characteristics of our sample. There were no significant differences between the treatment groups in age, BMI, race, income, education, marital status, ER/PR status, or stage of disease.

**TABLE 1:** Demographic and medical characteristics at baseline (*N* = 39).

Variable	BWL (*n* = 23)	DRL (*n* = 16)	*p* value*^a^*
Age: mean years (*SD*)	54.26 (9.31)	53.50 (8.96)	0.799
BMI (*SD*)	29.03 (7.78)	29.58 (8.25)	0.836
**Marital status: [*n* (%)]**			0.882
Never married	1 (4.4)	1 (6.3)	
Divorced	7 (30.4)	3 (18.8)	
Widowed	2 (8.7)	1 (6.3)	
Married	13 (56.5)	11 (68.8)	
**Ethnicity/race [*n* (%)]**			0.952
African American Black	4 (17.4)	2 (12.5)	
Asian	2 (8.7)	1 (6.3)	
Caucasian	15 (65.2)	13 (81.3)	
Other	2 (8.7)	0 (0.0)	
**Education [*n* (%)]**			0.879
Some high school or less	2 (8.7)	0 (0.0)	
Completed high school	6 (26.01)	6 (37.5)	
Some college	8 (34.8)	4 (25.0)	
College degree	7 (30.4)	6 (37.5)	
**Annual family income [*n* (%)]**			0.222
≤$15,000	5 (21.7)	3 (18.8)	
≤$30,000	6 (26.1)	0 (0.0)	
≤$50,000	1 (4.4)	2 (12.5)	
≤$100,000	4 (17.4)	2 (12.5)	
>$100,000	5 (21.7)	6 (37.5)	
Did not Answer	2 (8.7)	3 (18.8)	
**Menopausal status pre-chemotherapy [*n* (%)]**			0.982
Premenopausal	5 (21.7)	4 (25.0)	
Perimenopausal	3 (13.0)	2 (12.5)	
Postmenopausal	8 (34.8)	7 (43.8)	
Post-hysterectomy	6 (26.1)	3 (18.8)	
Unknown	1 (4.4)	0 (0.0)	
**Cancer stage [*n* (%)]**			0.789
Stage I	4 (17.4)	5 (31.3)	
Stage II	10 (43.5)	6 (37.5)	
Stage III	4 (17.4)	2 (12.5)	
Unknown	5 (21.7)	3 (18.8)	
**Surgery [*n* (%)]**			0.750
Lumpectomy	7 (30.4)	8 (50.0)	
Mastectomy	9 (39.1)	6 (37.5)	
Double mastectomy	4 (17.4)	1 (6.3)	
Pre-op chemotherapy	2 (8.7)	1 (6.3)	
Unknown	1 (4.4)	0 (0.0)	
**Chemotherapy regimen [*n* (%)]**			0.162
Exactly four cycles of AC	3 (13.0)	3 (18.8)	
Exactly four cycles of AC + Taxotere	5 (21.7)	0 (0.0)	
Exactly four cycles of AC + Taxol	6 (26.1)	2 (12.5)	
6 cycles of TAC	2 (8.7)	4 (25.0)	
Other regimen	4 (17.4)	6 (37.5)	
Unknown	3 (13.0)	1 (6.3)	
**Prior use of hormone replacement therapy [*n* (%)]**			0.155
Yes	2 (8.7)	4 (25.0)	
No	13 (56.5)	10 (62.5)	
Unknown	8 (34.8)	2 (12.5)	

*BWL, bright white light; DRL, dark red light; BMI, body mass index.*

*^a^Two sample T test for continuous variables and Fisher’s Exact test for categorical variables.*

### Objective Sleep Measures

#### Nighttime Sleep

At baseline, the BWL group had significantly less TST than the DRL group (*p* = 0.01), thus the baseline TST was adjusted in all linear mixed-effects models. No other group differences were found at baseline (both *p*’s > 0.2 for %sleep and TWT).

**FIGURE 3:**
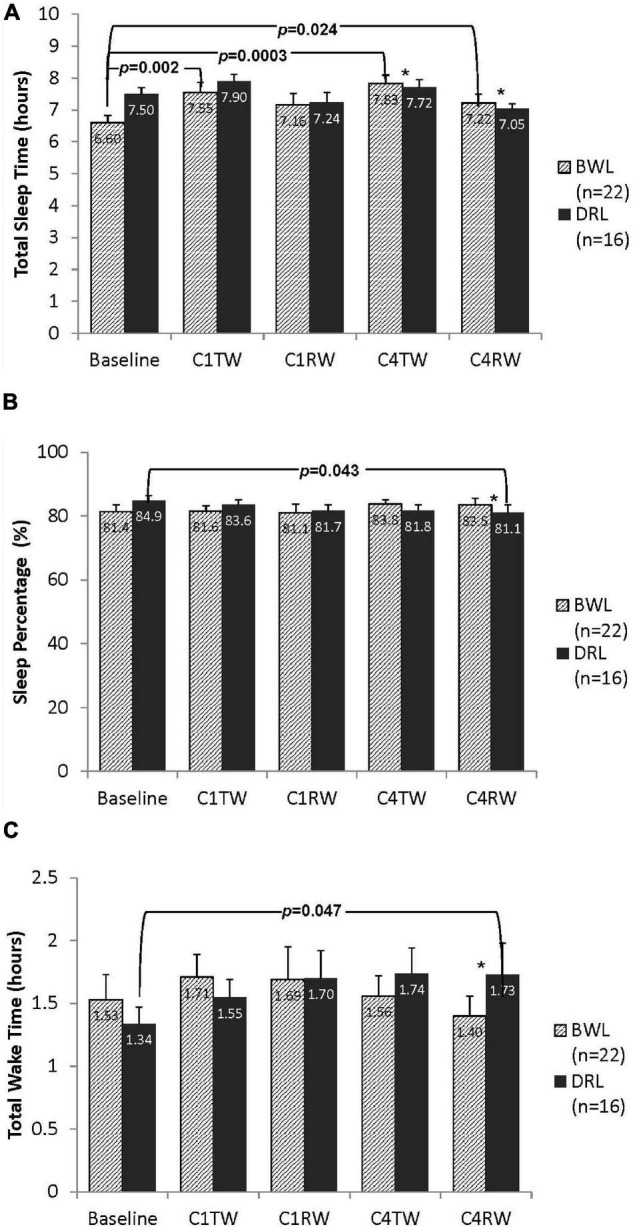
Bar graphs depicting nighttime (A) total sleep time, (B) sleep percentage, and (C) total wake time for both bright white light (BWL) and dim red light (DRL) treatment groups from baseline through the treatment weeks (TW) and recovery weeks (RW) of chemotherapy cycles 1 and 4. With the exception of recovery week of cycle 1 (C1RW), the BWL group demonstrated longer total sleep time (A) compared to baseline. On the other hand, DRL group demonstrated longer total wake time (C) and lower sleep percentage (B) during the recovery week of cycle 4 (C4RW). *p < 0.05 for group-by-time interaction, indicating that compared to DRL group, BWL group had significant longer total sleep time during cycle 4 (both C4TW and C4RW), significant higher sleep percentage and shorter total wake time during C4RW.

While controlling for baseline differences, a significant group-by-time interaction was found for TST at both C4TW (*p* = 0.042) and C4RW (*p* = 0.012; Figure 3A). Compared with baseline, the BWL group had significant increases in TST at C4TW and C4RW (*p*’s < 0.03), whereas the DRL group had no significant changes in TST at these time points.

Significant group-by-time interactions were found for %sleep and TWT at C4RW (Figures 3B,C). Compared with baseline, the DRL had a significant decrease in %sleep (*p* < 0.05) and a significant increase in TWT (*p* < 0.05) at C4RW, whereas the BWL had no significant changes in these variables at these time points.

#### Daytime Sleep

**FIGURE 4:**
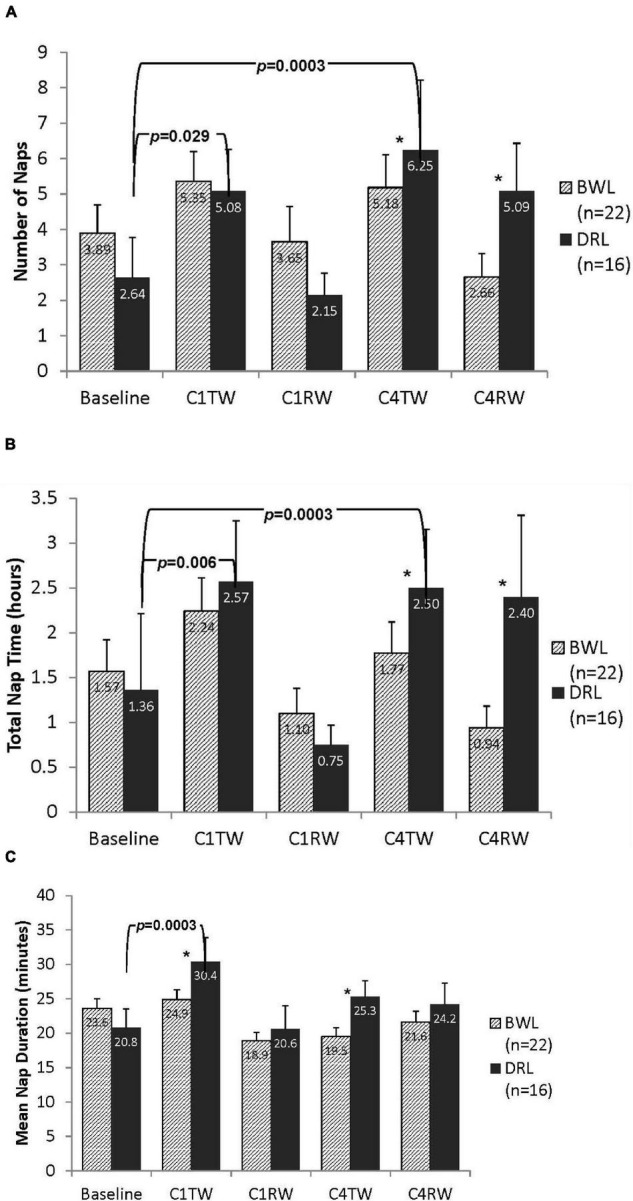
Bar graphs depicting daytime (A) number of naps, (B) total nap time, and (C) mean nap duration for both BWL and DRL treatment groups from baseline through the TW and RW of chemotherapy cycles 1 and 4. With the exception of recovery weeks (C1RW and C4RW), the DRL group demonstrated more frequent (A) and longer (B) naps as chemotherapy treatment progressed. Mean nap duration (C) also increased at C1TW for the DRL group, *p < 0.05 for group-by-time interaction, indicating that compared to DRL group, BWL group had significant fewer naps and shorter total nap time during cycle 4 (both C4TW and C4RW).

No group differences in daytime sleep at baseline were detected (all *p*’s > 0.3). A significant group-by-time interaction for nNAP was found at both C4TW and C4RW (*p’s* < 0.05; Figure 4A). Compared with baseline, nNAP increased significantly at C4TW (*p* < 0.03) and C4RW (*p* = 0.0003) in the DRL group, whereas the BWL group had no significant changes in nNAP at these time points.

Significant group-by-time interactions for TNT were found at C4TW and C4RW (*p*s < 0.05; Figure 4B). Compared with baseline, in the DRL group, TNT increased significantly at C4TW (*p* = 0.0003) and but changes during C4RW were not significant. In the BWL group there were no significant changes in TNT.

Significant group-by-time interactions for mNAP were found at C1TW (*p* < 0.03), C4TW (*p* < 0.05), and C4RW (*p* < 0.05; Figure 4C). Compared with baseline, mNAP increases significantly at C1TW (*p* < 0.0003) and C4TW (*p* < 0.05) and non-significantly at C4RW (*p* < 0.05) in the DRL group, whereas compared with baseline, mNAP had a small (non-significant) increase at C1TW, and small (non-significant) decreases at C4TW and C4RW in the BWL group.

### Activity

#### Activity During the Nighttime Sleep Period

**FIGURE 5:**
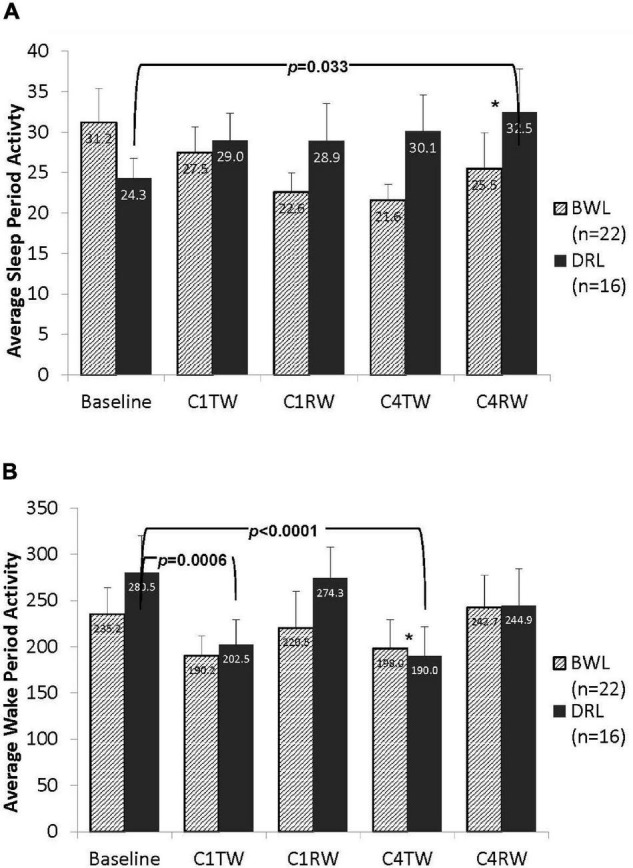
Bar graphs depicting average counts per minute for both (A) the nighttime sleep period and (B) the daytime wake period activity in the BWL and DRL treatment groups from baseline through the TW and RW of chemotherapy cycles 1 and 4. As depicted in panel (A), the average nighttime activity decreased in the BWL group while the DRL increased from baseline to the end of cycle 4 (C4RW). Conversely, as depicted in panel (B), the average daytime activity decreased in the DRL group from baseline to the treatment weeks of cycle 1 (C1TW) and cycle 4 (C4TW). *p < 0.05 for group-by-time interaction, indicating that compared to DRL group, BWL group had significant less daytime activity decrease during C4TW.

As shown in Figure 5A, activity counts during the nighttime sleep period did not differ between groups at baseline (*p* = 0.16). At C4RW a significant group-by-time interaction was found (*p* = 0.047). Compared with baseline, at C4RW the DRL group had a significant increase in nighttime sleep period activity (*p* = 0.033), whereas the BWL group showed a non-significant decrease in nighttime sleep activity count. No other group-by-time effect was found for activity during the night period.

#### Activity During the Daytime Wake Period

As shown in Figure 5B, activity counts during the daytime wake period did not differ between groups at baseline (*p* = 0.35). A significant group-by-time interaction was observed at C4TW (*p* = 0.013). Compared with baseline, at C4TW the DRL group had significantly less activity counts (*p* < 0.001), whereas the BWL did not have a significantly change in activity count. A similar pattern was observed during C1TW; compared with baseline, activity counts decreased in the DRL group (*p* < 0.001) but did not change significantly in the BWL group. However, the group-by-time interaction was not significant at C1TW, nor for other assessment times.

### Subjective Sleep Quality

Pittsburgh Sleep Quality Index global and component scores are listed in Table 2. There were no significant differences in the PSQI global or component scores between the BWL group and the DRL group at baseline (all *p*’s > 0.05), and also no significant group-by-time interactions for the global or component scores during either cycle (all *p*’s > 0.05). Within the BWL group, compared to baseline, there were significantly lower scores in three subscales (i.e., improvement in subjective sleep quality, sleep duration, sleep disturbances) during C4RW, however, the daytime dysfunction component score increased (i.e., worse daytime function) during both weeks of cycle 4 (both *p*’s < 0.05). Within the DRL, compared to baseline, the subjective sleep quality component score decreased during the recovery weeks of both cycles (i.e., sleep quality improved) but the use of sleeping medication increased during the treatment weeks of both cycles (all *p*’s < 0.05).

**TABLE 2:** Mean (SE) PSQI total and component scores by group condition and mixed model analysis.

PSQI	Bright white light					Dim red light				
		
	Baseline *N* = 22	C1TW *N* = 17	C1RW *N* = 18	C4TW *N* = 16	C4RW *N* = 15	Baseline *N* = 16	C1TW *N* = 14	C1RW *N* = 13	C4TW *N* = 14	C4RW *N* = 14
Global	8.9 (0.9)	9.1 (0.8)	8.6 (1.0)	8.5 (0.9)	6.9 (0.9)	7.9 (0.9)	8.1 (1.1)	7.1 (1.3)	7.9 (0.9)	6.9 (1.1)
Subjective sleep quality	1.3 (0.2)	1.4 (0.2)	1.0 (0.2)	0.9 (0.2)	0.4** (0.1)	1.6 (0.2)	1.4 (0.3)	1.0* (0.3)	1.1 (0.2)	0.9* (0.2)
Sleep latency	1.4 (0.2)	1.4 (0.2)	1.4 (0.2)	0.9 (0.3)	0.8 (0.3)	1.5 (0.3)	1.1 (0.3)	1.2 (0.3)	1.0 (0.3)	1.3 (0.3)
Sleep duration	1.3 (0.2)	0.9 (0.2)	0.9 (0.2	0.9 (0.2)	0.9* (0.2)	0.8 (0.1)	0.8 (0.2)	0.5 (0.2)	0.4 (0.2)	0.5 (0.1)
Habitual sleep efficiency	1.8 (0.3)	1.9 (0.3)	1.3 (0.3)	1.6 (0.3)	1.1 (0.3)	1.3 (0.3)	0.9 (0.3)	0.9 (0.3)	1.2 (0.4)	0.7 (0.3)
Sleep disturbances	1.5 (0.1)	1.4 (0.2)	1.2 (0.1)	1.3 (0.2)	1.1* (0.2)	1.6 (0.1)	1.5 (0.2)	1.4 (0.1)	1.7 (0.2)	1.5 (0.1)
Use of sleeping medication	1.1 (0.3)	1.4 (0.4)	1.7 (0.3)	1.8 (0.4)	1.6 (0.4)	0.7 (0.3)	1.5* (0.4)	1.3 (0.4)	1.4* (0.4)	1.2 (0.4)
Daytime dysfunction	0.6 (0.2)	0.8 (0.2)	0.9 (0.2)	1.1* (0.1)	1.0* (0.2)	0.6 (0.2)	0.9 (0.2)	0.7 (0.2)	1.0 (0.2)	0.8 (0.2)

Compared to Baseline in each group: *p < 0.05, **p < 0.01; there were no significant group by time interactions for both groups at any time point (all p’s > 0.1).

## Discussion

The results of this study suggest that morning bright white light administered daily during chemotherapy to women with breast cancer may help reduce deterioration of nighttime sleep and sleep quality and reduce daytime sleepiness.

During the weeks of chemotherapy administration, the weeks of greatest distress, the women in both treatment groups took more and longer naps. During the recovery week of cycle 1, both groups returned to the pre-chemotherapy levels. However, by the fourth cycle, the cumulative effects of chemotherapy resulted in less sleep at night and more and longer naps during the day in the women in the DRL group while women in the BWL group showed an increase in nighttime sleep and a return to pre-chemotherapy levels of napping.

Similar results were observed in sleep quality. While no significant group by time interaction was observed by the end of cycle 4 chemotherapy, compared to baseline, the BWL group reported improvement in nighttime sleep quality (subjective sleep quality, sleep duration and sleep disturbance components). Reports of daytime dysfunction, however, increased. This deterioration of daytime functioning during the fourth cycle of treatment might be attributed not only to disturbed sleep, but also to the cumulative side-effects of cancer treatment. The finding that the DRL group reported improved sleep quality during the treatment weeks of both cycles may be explained by the concurrent increase in sleep medication use (Huedo-Medina et al., 2013). Taken together, the objective sleep and subjective sleep quality results suggest that overall, the bright white light resulted in less deterioration of sleep.

In addition to the effects on sleep, significant changes in the amount of activity both during the sleep period and the wake period were observed. Berger et al. (2009) showed that there is little distinction between night and day activity, as measured by actigraphy during chemotherapy, which suggested both disrupted sleep at night and disrupted wake during the day (2009). Having high actigraphic activity counts during the wake period and low counts during the sleep period has been associated with higher survival (Mormont et al., 2000; Innominato et al., 2009), better quality of life (Innominato et al., 2009), and lower levels of depression (Du-Quiton et al., 2010) and fatigue (Innominato et al., 2009) in patients with cancer. Having a more robust circadian pattern of acigraphic activity and better sleep has also been predictive of less cognitive decline in women with breast cancer (Ancoli-Israel et al., 2021). In the current study, women exposed to dim red light had decreased wake-time activity during chemotherapy treatment weeks of cycle 1 and cycle 4, as might be expected during chemotherapy, while those exposed to bright white light had no significant changes in daytime activity compared to baseline. During the sleep period, those in the BWL group showed less activity than those in the DRL group. These data suggest that bright white light also protected the women from the deterioration in wake-time physical activity usually experienced during chemotherapy. Nonetheless, activity levels during the wake period are considered low (Kwan et al., 2020). Even modest increases in physical activity could potentially elicit improvements in sleep (Mercier et al., 2017) and in circadian synchronization (Youngstedt et al., 2019).

The impetus for this study was the prior observation that women undergoing chemotherapy receive progressively less bright light exposure as treatment progresses, particularly in the days following chemotherapy infusion, and that this decrease is associated with fatigue and sleep disturbances (Liu et al., 2005). Previously reported data from this sample demonstrated that bright light therapy prevented cancer-related fatigue (Ancoli-Israel et al., 2011) and prevented deterioration of both the circadian activity rhythm (Neikrug et al., 2012) and subjective quality of life during chemotherapy (Jeste et al., 2013). The current results demonstrate not only a lack of deterioration of sleep and activity in the bright white light group but also improvement in daytime sleep and nighttime activity compared to pre-chemotherapy levels.

We believe that the most likely mechanisms mediating sleep improvement reported in the present study are the indications of better circadian entrainment by light (Neikrug et al., 2012). Bright light entrains the circadian system by stimulating retinal photoreceptors in the retina, which interact with the SCN *via* a monosynaptic pathway, as well as multi-synaptic pathways from the retina to the ventral lateral geniculate nucleus and intergeniculate nucleus (Golombek and Rosenstein, 2010). Effects of bright light on alertness and sleepiness (Badia et al., 1991) also might have contributed to better nighttime sleep, as well as reduced napping noted in the present study. However, whether the observed benefit of bright light therapy was due to the alerting effect of light, to the improvement in circadian activity rhythms or some other unobserved mechanism, cannot be determined from this study.

While there have been a few other studies examining the effect of bright light treatment on sleep in cancer survivors (Johnson et al., 2016, 2018; Starreveld et al., 2018; Valdimarsdottir et al., 2018; Wu et al., 2018; Fox et al., 2020), to our knowledge, this is the first randomized controlled trial examining the effect of bright light therapy on sleep (measured both objectively and subjectively) and activity during chemotherapy in women with breast cancer. Berger et al. (2009) found a positive effect on sleep in a similar population using a modified behavioral therapy that included both nighttime and daytime sleep restriction; however, the improvement in the treatment group was limited to an improvement in subjective sleep quality and fewer objectively measured awakenings at night (daytime sleep was not reported). While these results are suggestive that targeting both nighttime and daytime sleep disruption using a behavioral treatment may be beneficial during chemotherapy, the lack of objective improvement in sleep also suggests that additional intervention may be needed.

Notwithstanding the significant group-by-time effects for several of the variables, the clinical significance of some of these effects was minimal. While bright light has been shown to be highly effective in fatigue, circadian rhythms and quality of life in these same women (Ancoli-Israel et al., 2011; Neikrug et al., 2012; Jeste et al., 2013), the percent sleep remained low and total wake time remained high at all time points. Combining bright light with other treatments, such as cognitive behavioral treatment for insomnia (Bean et al., 2020) or exercise (Mercier et al., 2017) may have additive benefits with greater clinical efficacy.

The strengths of the current study include the randomized controlled and longitudinal design; in particular, the inclusion of a baseline prior to chemotherapy in addition to data collection during chemotherapy. Additional strengths include the inclusion of both subjective and objective measures of sleep and the utilization of the mixed model statistical analysis which allowed for partially complete subject records (i.e., missing data at some time-points), thereby avoiding the biases of “completers only” analysis.

However, there are also limitations to the study. The first major limitation is the small sample size. With a larger sample size, trends such as the deterioration found in the DRL group may have been statistically significant. However, this was a preliminary study intended to provide Phase II data for a larger randomized trial. Secondly, the physical activity results should be interpreted with caution as we did not employ waist actigraphy. Our main interest was on sleep for which wrist actigraphy is a reliable measure (Ancoli-Israel et al., 2003; Ancoli-Israel et al., 2015). More detailed assessment of physical activity and exercise is needed in future work. A third limitation may be the 72-h period of actigraphy data collection. This shorter period was chosen to both reduce patient burden and to ensure sufficient time for baseline data collection as often the time period between recruitment and the start of chemotherapy was less than 1 week.

In summary, the breast cancer chemotherapy group receiving dim red light showed expected and progressive deterioration during chemotherapy, particularly in daytime sleepiness and inactivity during the day during cycle 4. The bright white light group, however, showed significantly less deterioration and were less sleepy and more active during the day at cycle 4 showing a greater ability to recover from the cumulative negative effects of chemotherapy. Larger studies are needed to replicate these findings; however, the study suggests that morning bright light, an easy, non-invasive, non-harmful behavioral treatment, may at least prevent deterioration of nighttime sleep and promote daytime activity and alertness in women undergoing chemotherapy for breast cancer.

## Data Availability Statement

The raw data supporting the conclusions of this article will be made available by the authors, without undue reservation.

## Ethics Statement

The studies involving human participants were reviewed and approved by University of California San Diego Office of IRB Administration and by the UC San Diego Moores Cancer Center’s Protocol Review and Monitoring Committee. The patients/participants provided their written informed consent to participate in this study.

## Author Contributions

SA-I was the PI of the study. MR led the writing of the manuscript and contributed to the data collection and interpretation. LL contributed to the study conception, design, material preparation, and commenting on all versions of the manuscript. SY contributed to writing and commenting on previous versions of the manuscript. VT and LN contributed to the study conception, design, material preparation, and writing and commenting on previous versions of the manuscript. LN performed the statistical analyses. AN contributed to material preparation, data collection, and commenting on all versions of the manuscript. SA-I, NJ, and BP contributed to the study conception, design, material preparation, and writing and commenting on all versions of the manuscript. All authors read and approved the final manuscript.

## Conflict of Interest

SA-I was a consultant for Eisai, Biogen, Merck, Idorsia, and Pear Therapeutics. NJ was a student at UCSD at the time of the study and currently works for J&J which has had no influence or funding of this study. The remaining authors declare that the research was conducted in the absence of any commercial or financial relationships that could be construed as a potential conflict of interest.

## Publisher’s Note

All claims expressed in this article are solely those of the authors and do not necessarily represent those of their affiliated organizations, or those of the publisher, the editors and the reviewers. Any product that may be evaluated in this article, or claim that may be made by its manufacturer, is not guaranteed or endorsed by the publisher.

## Footnotes

1
http://cran.r-project.org/
