# Corrigendum: Preventing sleep disruption with bright light therapy during chemotherapy for breast cancer: a phase II randomized controlled trial

**DOI:** 10.3389/fnins.2024.1386314

**Published:** 2024-10-03

**Authors:** Michelle Rissling, Lianqi Liu, Shawn D. Youngstedt, Vera Trofimenko, Loki Natarajan, Ariel B. Neikrug, Neelum Jeste, Barbara A. Parker, Sonia Ancoli-Israel

**Affiliations:** ^1^Department of Psychiatry, University of California, San Diego, San Diego, CA, United States; ^2^Department of Medicine, University of California, San Diego, San Diego, CA, United States; ^3^Edson College of Nursing and Health Innovation, Arizona State University, Phoenix, AZ, United States; ^4^St. Joseph Hospital, Orange, CA, United States; ^5^Family Medicine and Public Health, University of California, San Diego, San Diego, CA, United States; ^6^Moores Cancer Center, University of California, San Diego, San Diego, CA, United States; ^7^Department of Psychiatry and Human Behavior, University of California, Irvine, Irvine, CA, United States; ^8^Johnson & Johnson, San Diego, CA, United States

**Keywords:** breast cancer, light therapy, sleep, actigraphy, PSQI, activity

In the published article, there was an error in Table 2 as published. The sample size numbers were reversed. The Bright White Light Group sample size numbers in the original table were really the sample sizes for the Dim Red Light group, and visa versa. The correct sample size numbers appear in the corrected table. The corrected Table 2 and its caption appear below.

**Table 2:** Mean (SE) PSQI total and component scores by group condition and mixed model analysis.

**PSQI**	**Bright white light**					**Dim red light**				
	**Baseline** ***N*** = **22**	**C1TW** ***N*** = **17**	**C1RW** ***N***= **18**	**C4TW** ***N*** = **16**	**C4RW** ***N*** = **15**	**Baseline** ***N*** = **16**	**C1TW** ***N*** = **14**	**C1RW** ***N*** = **13**	**C4TW** ***N*** = **14**	**C4RW** ***N*** = **14**
Global	8.9 (0.9)	9.1 (0.8)	8.6 (1.0)	8.5 (0.9)	6.9 (0.9)	7.9 (0.9)	8.1 (1.1)	7.1 (1.3)	7.9 (0.9)	6.9 (1.1)
Subjective sleep quality	1.3 (0.2)	1.4 (0.2)	1.0 (0.2)	0.9 (0.2)	0.4^**^ (0.1)	1.6 (0.2)	1.4 (0.3)	1.0^*^ (0.3)	1.1 (0.2)	0.9^*^ (0.2)
Sleep latency	1.4 (0.2)	1.4 (0.2)	1.4 (0.2)	0.9 (0.3)	0.8 (0.3)	1.5 (0.3)	1.1 (0.3)	1.2 (0.3)	1.0 (0.3)	1.3 (0.3)
Sleep duration	1.3 (0.2)	0.9 (0.2)	0.9 (0.2	0.9 (0.2)	0.9^*^ (0.2)	0.8 (0.1)	0.8 (0.2)	0.5 (0.2)	0.4 (0.2)	0.5 (0.1)
Habitual sleep efficiency	1.8 (0.3)	1.9 (0.3)	1.3 (0.3)	1.6 (0.3)	1.1 (0.3)	1.3 (0.3)	0.9 (0.3)	0.9 (0.3)	1.2 (0.4)	0.7 (0.3)
Sleep disturbances	1.5 (0.1)	1.4 (0.2)	1.2 (0.1)	1.3 (0.2)	1.1^*^ (0.2)	1.6 (0.1)	1.5 (0.2)	1.4 (0.1)	1.7 (0.2)	1.5 (0.1)
Use of sleeping medication	1.1 (0.3)	1.4 (0.4)	1.7 (0.3)	1.8 (0.4)	1.6 (0.4)	0.7 (0.3)	1.5^*^ (0.4)	1.3 (0.4)	1.4^*^ (0.4)	1.2 (0.4)
Daytime dysfunction	0.6 (0.2)	0.8 (0.2)	0.9 (0.2)	1.1^*^ (0.1)	1.0^*^ (0.2)	0.6 (0.2)	0.9 (0.2)	0.7 (0.2)	1.0 (0.2)	0.8 (0.2)

Compared to Baseline in each group: ^*^p < 0.05, ^**^p < 0.01; there were no significant group by time interaction for both groups at any time point (all p's > 0.1).

The authors apologize for this error and state that this does not change the scientific conclusions of the article in any way. The original article has been updated.

